# ^111^In-BnDTPA-F3: an Auger electron-emitting radiotherapeutic agent that targets nucleolin

**DOI:** 10.1186/2191-219X-2-9

**Published:** 2012-02-20

**Authors:** Bart Cornelissen, Andrew Waller, Carol Target, Veerle Kersemans, Sean Smart, Katherine A Vallis

**Affiliations:** 1 Department of Oncology, Cancer Research UK/Medical Research Council Gray Institute for Radiation Oncology and Biology, University of Oxford, Old Road Campus Research Building, Off Roosevelt Drive, Oxford, OX3 7DQ, UK

**Keywords:** F3 peptide, Auger electron, nucleolin, nucleolus, ^111^In.

## Abstract

**Introduction:**

The F3 peptide (KDEPQRRSARLSAKPAPPKPEPKPKKAPAKK), a fragment of the human high mobility group protein 2, binds nucleolin. Nucleolin is expressed in the nuclei of normal cells but is also expressed on the membrane of some cancer cells. The goal was to investigate the use of ^111^In-labeled F3 peptide for Auger electron-targeted radiotherapy.

**Methods:**

F3 was labeled with fluorescein isothiocyanate (FITC) for confocal microscopy and conjugated to p-SCN-benzyl-diethylenetriaminepentaacetic acid (BnDTPA) for labeling with ^111^In to form ^111^In-BnDTPA-F3. MDA-MB-231-H2N (231-H2N) human breast cancer cells were exposed to ^111^In-BnDTPA-F3 and used in cell fractionation, γH2AX immunostaining (a marker of DNA double-strand breaks), and clonogenic assays. *In vivo*, biodistribution studies of ^111^In-BnDTPA-F3 were performed in 231-H2N xenograft-bearing mice. In tumor growth delay studies, ^111^In-BnDTPA-F3 (3 μg, 6 MBq/μg) was administered intravenously to 231-H2N xenograft-bearing mice once weekly for 3 weeks.

**Results:**

Membrane-binding of FITC-F3 was observed in 231-H2N cells, and there was co-localization of FITC-F3 with nucleolin in the nuclei. After exposure of 231-H2N cells to ^111^In-BnDTPA-F3 for 2 h, 1.7% of ^111^In added to the medium was membrane-bound. Of the bound ^111^In, 15% was internalized, and of this, 37% was localized in the nucleus. Exposure of 231-H2N cells to ^111^In-BnDTPA-F3 (1 μM, 6 MBq/μg) resulted in a dose-dependent increase in γH2AX foci and in a significant reduction of clonogenic survival compared to untreated cells or cells exposed to unlabeled BnDTPA-F3 (46 ± 4.1%, 100 ± 1.8%, and 132 ± 7.7%, respectively). *In vivo*, tumor uptake of ^111^In-BnDTPA-F3 (3 μg, 6 MBq/μg) at 3-h post-injection was 1% of the injected dose per gram (%ID/g), and muscle uptake was 0.5%ID/g. In tumor growth delay studies, tumor growth rate was reduced 19-fold compared to untreated or unlabeled BnDTPA-F3-treated mice (*p *= 0.023).

**Conclusion:**

^111^In-BnDTPA-F3 is internalized into 231-H2N cells and translocates to the nucleus. ^111^In-BnDTPA-F3 has a potent cytotoxic effect *in vitro *and an anti-tumor effect in mice bearing 231-H2N xenografts despite modest total tumor accumulation.

## Introduction

Targeted radiotherapy uses alpha-, beta-, or Auger electron emissions from radionuclides to specifically irradiate cancer cells and cause tumor growth arrest. Well-established examples include the FDA-approved beta-particle-emitting drugs Bexxar (^131^I-tositumomab) and Zevalin (^90^Y-ibritumomab tiuxetan) for the treatment of CD20-positive non-Hodgkin's lymphoma [1]. Auger electron radiation therapy differs from alpha and beta particle therapy because of the extremely short pathlength of the low-energy electrons that are emitted [2-7]. Cellular internalization and subsequent nuclear localization are deemed necessary for Auger electron radiation to be effective for cancer therapy [8]. The tumor-homing peptide F3, a 31-mer peptide (KDEPQRRSARLSAKPAPPKPEPKPKKAPAKK), derived from human high mobility group protein 2 (HMGN2) has been shown to bind specifically to nucleolin expressed on the membrane of cancer cells, neovasculature, and endothelium. After binding, F3 internalizes into the targeted cell and translocates to the nucleus [9,10]. Zhang et al. targeted MCF7 cells using F3-conjugated dextran-coated iron oxide nanoparticles [11]. F3 peptide has also been used for the delivery of photodynamic therapy agents, iron oxide nanoparticles, siRNA, and oligonucleotides to various tumor cell lines and tumor xenografts [12-15]. Winer et al. have recently demonstrated targeting of F3-conjugated cisplatin-hydrogel nanoparticles to the vessels of various tumor xenografts [16]. Bhojani et al. and Van Dort et al. have recently reported on the use of radioiodine-labeled F3 peptide for single-photon emission computed tomography (SPECT) imaging [17,18]. A F3-peptide dimer labeled with the alpha-emitter ^213^Bi (^213^Bi-(F3)_2_) for molecular radiotherapy of membranar-nucleolin-expressing MDA-MB-435 cells was shown to be effective *in vitro *and in xenografts in mice [19]. When F3 was labeled with ^67^Ga, it accumulated in MDA-MB-435 tumor xenografts *in vivo*, as shown by PET imaging [19].

In the current report, an Auger electron-emitting, ^111^In-labeled (*t*_1/2 _= 2.9 days), benzyl-diethylenetriaminepentaacetic acid (BnDTPA) conjugated monovalent variant of the F3 peptide is described. Auger electrons are low-energy and are emitted by radionuclides that decay by electron capture. Their very short pathlength (approximately 1 nm to 1 μm), but with high linear energy transfer-like characteristics, means that they can cause irreparable and cell-lethal clustered DNA damage when they are emitted inside the nucleus, in close proximity to DNA. Here, we show that ^111^In-BnDTPA-F3 internalizes into the cells and translocates to the nuclei and nucleoli, and that the Auger electron emission causes DNA double-strand breaks (DNA dsb) and cytotoxicity. The clonogenic survival of cells exposed to ^111^In-BnDTPA-F3, its biodistribution in xenograft-bearing mice, and ability to arrest tumor xenograft growth are reported.

## Materials and methods

### Cell lines

MDA-MB-231 cells, stably transfected with the *HER2 *gene yielding MDA-MB-231-H2N cells (referred to as 231-H2N), were a gift from Dr. R. Kerbel (Sunnybrook Health Sciences Centre, Toronto, Ontario, Canada) [20]. Cells were cultured at 37°C in 5% CO_2 _using Dulbecco's modified eagle medium (DMEM) cell culture medium (Sigma-Aldrich, Dorset, UK) supplemented with a 10% fetal calf serum (Invitrogen, Paisley, UK) and penicillin/streptomycin, 100 units/mL (Invitrogen, Paisley, UK).

### Synthesis of ^111^In-BnDTPA-F3

F3 peptide (KDEPQRRSARLSAKPAPPKPEPKPKKAPAKK, MW = 3432 g/mol) was obtained from Cambridge peptides (Cambridge, UK). Size and purity were confirmed by reverse phase HPLC and mass spectroscopy. F3 peptide (100 μg dissolved in 100 μL 0.1 M sodium bicarbonate, pH 8.4) was reacted with a fivefold molar excess of p-SCN-BnDTPA (Macrocyclics, Dallas, TX, USA), dissolved in dry DMSO (Sigma, Gillingham, Dorset, UK) at room temperature for 1 h, resulting in BnDTPA-F3. Unconjugated BnDTPA was removed by centrifugation using a YM-3 microfilter (Millipore, Billerica, MA, USA). BnDTPA-F3 peptide was buffer-exchanged in 0.1 M sodium citrate pH 5.0. The DTPA conjugation rate was determined as previously described by Hnatowich et al. [21] and was found to be 0.85:1 to 0.95:1 DTPA/F3. ^111^In chloride (0.1 to 9 MBq/μg, 0.34 to 30.9 MBq/nmol) (Perkin Elmer, Waltham, MA, USA) was added, resulting in ^111^In-BnDTPA-F3. After incubation at room temperature for 1 h, radiolabeling yield was measured using instant thin layer chromatography (Amersham Health, Amersham, UK) in 0.1 M sodium citrate pH 5.0 and was always ≥ 95%.

### Confocal microscopy

The 231-H2N cells were seeded on coverslips and allowed to adhere overnight. Cells were exposed to 10-nM fluorescein isothiocyanate (FITC)-conjugated F3 peptide (Phoenix Pharmaceuticals, Karlsruhe, Germany) for 2 h at 37°C. Cells were washed with phosphate buffered saline (PBS), fixed for 10 min at room temperature with 4% paraformaldehyde (Sigma-Aldrich Corporation, St. Louis, MO, USA), permeabilized at room temperature using 1% Triton X-100 in PBS (Sigma), and blocked (1 h at 37°C, 2% BSA in PBS). Cells were immunostained for nucleolin using mouse anti-nucleolin IgG as primary antibody for 1 h at 37°C (1:2,000, ab13541; Abcam, Cambridge, UK), incubated with Alexa Fluor 555 goat anti-mouse IgG (1:250; Invitrogen, Paisley, UK), and mounted using Vectashield mounting medium with DAPI (Vector labs, Peterborough, UK). Confocal microscopy images were acquired using a Zeiss 530 microscope (Zeiss, Welwyn Garden City, UK).

### Internalization and nuclear localization of ^111^In-BnDTPA-F3

Internalization and retention of ^111^In-BnDTPA-F3 were determined as previously described by Cornelissen et al. [22]. Briefly, 2 × 10^5 ^cells were seeded per well in 24-well plates, and ^111^In-BnDTPA-F3 was then added (1 μM in 200 μL DMEM, 6 MBq/μg). For internalization assays, the supernatant was removed at selected time points, and cells were washed using 0.1 M glycine HCl pH 2.5 to remove cell-surface bound ^111^In-BnDTPA-F3. Cells were then lyzed using 0.1 M NaOH. Radioactivity in fractions containing supernatant, cell-surface bound, and internalized radiopeptide was counted using an automated gammacounter system (Wizard, PerkinElmer, Waltham, MA, USA). In some cases, a 100-fold molar excess of cold, unlabeled F3 or anti-nucleolin antibody (ZN004; MBL International, Woburn, MA, USA) was added. ZN004 has been shown to block the uptake of fluorescently labeled F3 peptide by inhibiting internalization of nucleolin [13]. For nuclear localization assays, aliquots of 10^6 ^cells in 500 μL of growth medium were exposed to ^111^In-BnDTPA-F3 (1 μM in 200 μL DMEM, 6 MBq/μg). At selected time points, the supernatant was removed, and cells were washed with 0.1 M glycine HCl pH 2.5 to remove cell-surface bound F3. The cell membrane was lysed (25 mM KCl, 5 mM MgCl_2_, 10 mM Tris-HCl, and 0.5% NP-40; 6 min on ice) [22,23]. The cytoplasmic fraction was separated by centrifugation. Nuclei were washed with PBS and lysed using 0.1 M NaOH. Radioactivity in the fractions containing supernatant, cell-surface bound, cytoplasmic, and nucleus-associated ^111^In was counted using an automated gammacounter system. We previously demonstrated by Western blot for calpain, an abundant cytoplasmic protein and p84, a nuclear matrix protein (unpublished results), that this method provides a highly pure nuclear and cytoplasm/membrane fraction.

### Microdosimetry

Using the data obtained from nuclear localization assays, time-activity curves were generated for each of the compartments of the cell: membrane, cytoplasm, and nucleus. Using *S *values tabulated by Goddu et al., the radiation dose delivered to the nucleus of 231-H2N cells was calculated [24].

### γH2AX assay

The 231-H2N cells (2 × 10^5^) were seeded in 96-well plates and left to adhere overnight. Fresh growth medium (2 mL) containing various amounts (0 to 5 μM) of cold, unlabeled BnDTPA-F3 or ^111^In-BnDTPA-F3 (6 MBq/μg) was added. X-ray irradiated cells (4 Gy) were used as a positive control. After incubation at 37°C for 2 or 24 h, cells were washed, fixed, permeabilized, and blocked. Samples were stained for γH2AX as described previously by Nakamura et al. [25]. Fluorescence images were acquired using an IN Cell analyzer (GE Healthcare, Pittsburgh, PA, USA). The number of γH2AX foci per cell was counted automatically using the IN Cell analysis software. In a separate experiment, cells were exposed to BnDTPA-F3 (2 μM) at increasing specific activities (0 to 6 MBq/μg or 0 to 20.6 MBq/nmol) for 2 h, and the number of γH2AX foci was determined as above.

### Clonogenic survival

Aliquots of 231-H2N cells (2 × 10^5^) were exposed to various concentrations (range, 0 to 1 μM) of cold, unlabeled BnDTPA-F3 or ^111^In-BnDTPA-F3 (6 MBq/μg). After incubation at 37°C for 24 h, 2 × 10^3 ^cells were seeded per well in a 6-well plate, and 2 mL of fresh growth medium was added. After 7 to 14 days, cell colonies were stained with methylene blue (2% methylene blue in water/methanol 1:1) and counted. Combination indices (CI) of ^111^In with BnDTPA-F3 were determined as described by Edelman et al. [26]. A CI < 0.9 indicates superadditivity.

### *In vivo *biodistribution and SPECT imaging

All animal procedures were carried out in accordance with the United Kingdom Animals (Scientific Procedures) Act 1986 and with the local ethical committee approval. The 231-H2N xenografts were established in female balb/c *nu/nu *mice (Harlan, UK) by subcutaneous injection in the right flank of 5 × 10^6 ^cells in DMEM/matrigel 1:1 (Matrigel; BD Biosciences, Oxford, UK). When xenografts reached a volume of approximately 500 μL, ^111^In-BnDTPA-F3 (3 μg, 6 MBq/μg) was administered to the mice intravenously (i.v.) (*n *= 3). Preliminary kinetic planar gamma imaging showed that a plateau for tumor uptake was reached after 3 h (data not shown). Therefore, at 3 h after injection, static SPECT images were acquired using a nanoSPECT/CT system (Bioscan, Washington DC, USA). After SPECT imaging, animals were euthanized, and blood, selected normal tissues, and tumor were removed. Tissues were washed in PBS and blot dried, weighed, and counted for radioactivity. The amount of ^111^In in blood and tissues was expressed as a percentage of the injected dose per gram (%ID/g) of blood/tissue.

### Tumor growth inhibition

The 231-H2N xenografts were established in female balb/c *nu/nu *mice as described above. When xenografts reached a volume of approximately 100 μL, PBS (control), unlabeled F3 peptide or ^111^In-BnDTPA-F3 (3 μg, 6 MBq/μg) was administered i.v. to the mice (seven per group) on days 1, 8, and 15. Tumor size was measured by a caliper twice weekly. Tumor volume was calculated as *V *= *a*^2 ^× *b*, where a and b are the short and long axes, respectively. Animals were euthanized when the tumor diameter reached 12.5 mm. Tumor growth rate (*K*) was estimated by fitting the volume data to *V *= *V*_0 _× *e^k × t^*, where *V *is the tumor volume in mm^3^, *V_0 _*is the volume at the start of the experiment, *t *is the time after treatment in days, and *K *is the growth rate expressed in mm^3^/day.

### Statistical analysis

All statistical analyses were performed using GraphPad Prism (GraphPad Software, Inc., La Jolla, CA, USA). Data are reported as mean ± standard deviation of at least three independent replicates throughout, except for γH2AX foci analysis, where the results are expressed as mean ± standard error of at least 200 cells per condition. One-way or two-way ANOVA was used for multiple comparisons. Tukey post-tests were used after one-way ANOVA. The *F*-test was used to compare fitted curves. Kaplan-Meier curves were generated for survival analysis. Log-rank tests were performed to compare Kaplan-Meier survival curves.

## Results

### Intracellular distribution of FITC-F3 and ^111^In-BnDTPA-F3

**Figure 1:**
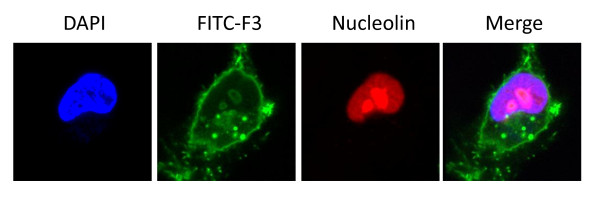
**FITC-F3 confocal microscopy**. The 231-H2N cells were exposed to fluorescein isothiocyanate (FITC)-F3 (10 nM) for 2 h. FITC-F3 is shown in green and nucleolin in red. Nuclei were stained with DAPI (blue). Images of a representative cell are shown.

**Figure 2:**
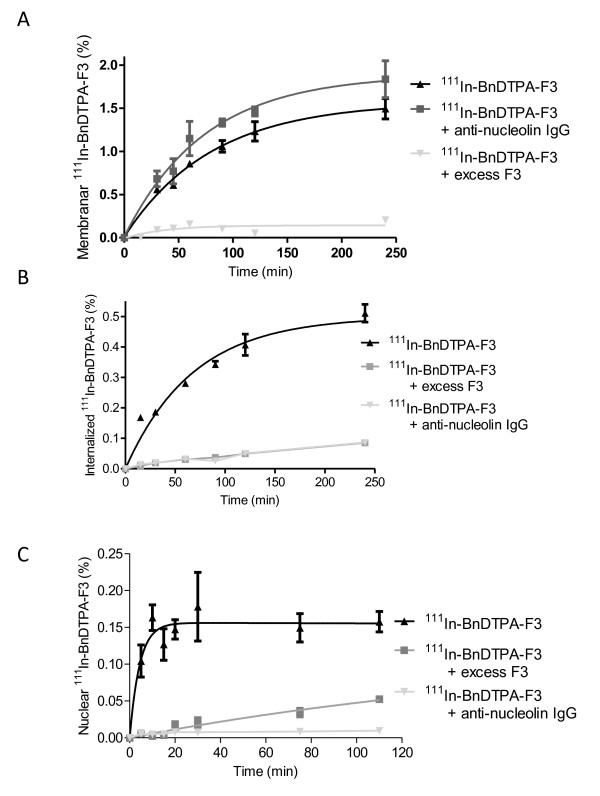
**Cellular and nuclear uptake of ^111^In-BnDTPA-F3**. The 231-H2N cells were exposed to ^111^In-benzyl-diethylenetriaminepentaacetic acid (BnDTPA)-F3 alone or in combination with a 100-fold molar excess of cold, unlabeled BnDTPA-F3 or anti-nucleolin antibody for various times. (**A**) Membranar fraction, (**B**) cellular internalization, and (**C**) nuclear localization of ^111^In were determined. Results are expressed as mean ± SD of three independent repeats.

Confocal microscopy showed membrane-bound and internalized fluorescence in 231-H2N cells after exposure to FITC-F3 for 2 h. FITC-F3 was observed to be present in nuclei. Based on density measurements, nuclear localization of FITC-F3 was approximately 33.6% of the total amount internalized. There, it co-localized with nucleolin in nucleoli. Representative confocal images of the intracellular distribution of FITC-F3 and nucleolin in 231-H2N cells are shown in Figure 1. Cell fractionation experiments were performed to evaluate the extent of cellular and nuclear uptake of ^111^In-BnDTPA-F3 in 231-H2N cells. After exposure of 231-H2N cells to 1-μM ^111^In-BnDTPA-F3 for 2 h, 0.51 ± 0.03% of the total added ^111^In was internalized (Figure 2B). When an excess of cold, unlabeled F3 or anti-nucleolin antibody was added, internalization of ^111^In-BnDTPA-F3 was decreased significantly (*p *< 0.001). Of the internalized fraction of ^111^In-BnDTPA-F3, 37% translocated to the nuclei, similar to the 33.6% found for FITC-F3. Nuclear localization plateaued after 30 min at values of 0.15 ± 0.05% (Figure 2C). Using these data, the radiation absorbed dose to 231-H2N cells was calculated using *S *value tables [24]. It was estimated that the exposure of 231-H2N cells to 1-μM ^111^In-BnDTPA-F3 (6 MBq/μg) resulted in a radiation absorbed dose 10.8 Gy over 2 h. The contributions from the membrane, cytoplasm, and nucleus were 2.3, 1.3, and 7.1 Gy, respectively.

### γH2AX assay

**Figure 3:**
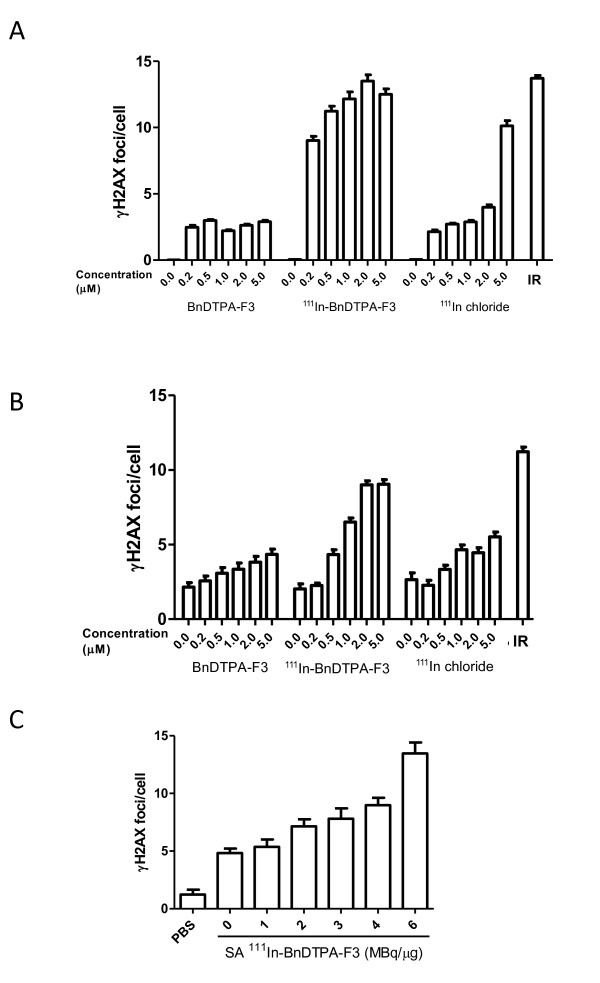
**Induction of DNA double-strand breaks by ^111^In-BnDTPA-F3**. The 231-H2N cells were X-irradiated (IR, 4 Gy) or exposed to ^111^In-BnDTPA-F3 (0 to 5 μM, 20.6 MBq/nmol), an equivalent amount of ^111^In chloride, or cold, unlabeled BnDTPA-F3 for (**A**) 2 h or (**B**) 24 h. Cells were fixed and stained for γH2AX. The number of γH2AX foci per cell was determined. Results are expressed as mean ± SEM, *n *= 200 per condition.(**C**) The 231-H2N cells were exposed to ^111^In-BnDTPA-F3 (2 μM) with varying specific activities (0 to 6 MBq/μg) for 2 h and treated as above.

The number of γH2AX foci, a measure for the number of DNA dsb, increased slightly after a 2-h exposure of 231-H2N cells to cold, unlabeled BnDPTA-F3. After incubation with BnDPTA-F3 for 24 h, the number of γH2AX foci in 231-H2N cells did not change significantly. However, the number of γH2AX foci per cell increased in a dose-dependent manner in 231-H2N cells that were exposed to ^111^In-BnDTPA-F3, following incubation for 2 or 24 h (*F*-test, *p *< 0.001) (Figure 3A, B). Irradiated cells (4 Gy), used as a positive control, had significantly more foci/cell compared with the untreated control cells (*p *< 0.0001). The number of γH2AX foci 2 h after irradiation (4 Gy) was not significantly different from that after ^111^In-BnDTPA-F3 (6 MBq/μg) at concentrations higher than 0.2 μM (*p *> 0.05). Furthermore, γH2AX foci induction was linearly dependent of the specific activity used (Spearman, *R *= 0.99; *p *= 0.0028).

### Clonogenic survival assay

**Figure 4:**
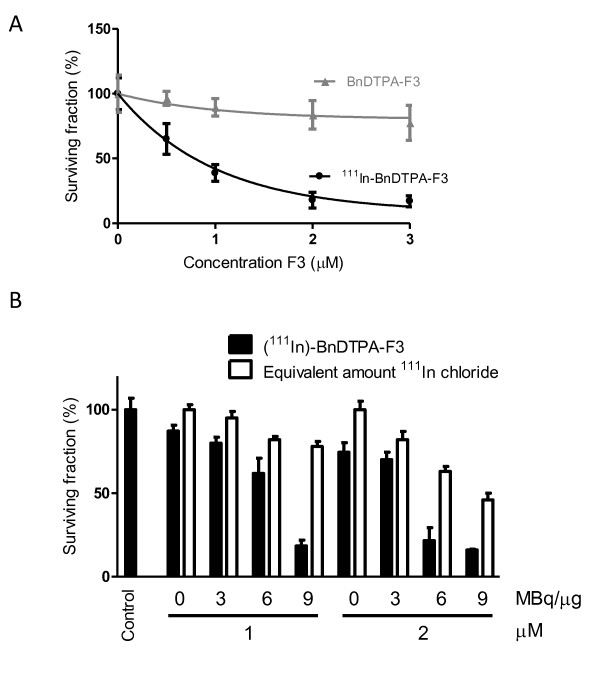
**Clonogenic survival of cells after exposure to ^111^In-BNDTPA-F3**. **(A) **Clonogenic survival of 231-H2N cells, exposed for 24 h to increasing concentrations (0 to 3 μM) of ^111^In-BnDTPA-F3 (20.6 MBq/nmol), or cold, unlabeled BnDTPA-F3. **(B) **Clonogenic survival of 231-H2N cells, exposed for 24 h to increasing concentrations (0 to 2 μM) of ^111^In-BnDTPA-F3, at increasing specific activities (0 to 30.9 MBq/nmol), or equivalent amounts of ^111^In chloride. Results are expressed as mean ± SD of three independent repeats.

Clonogenic survival of 231-H2N cells was reduced significantly by increasing concentrations of ^111^In-BnDTPA-F3 (Figure 4A). The surviving fraction was reduced to 10% after exposure to ^111^In-BnDTPA-F3 (3 μM, 6 MBq/μg) for 24 h (*p *< 0.001). In contrast, exposure to cold, unlabeled BnDTPA-F3 did not significantly reduce clonogenic survival. To evaluate the effect of increased specific activity, clonogenic survival of 231-H2N cells was also evaluated after exposure to ^111^In-BnDTPA-F3 (up to 2 μM) of increasing specific activity ranging from 0 to 9 MBq/μg (Figure 4B). The increasing specific activity of a 2-μM amount of ^111^In-BnDTPA-F3 resulted in a 4.6-fold decrease in clonogenic survival from 74.5 ± 5.8% to 16 ± 0.5% compared with the unexposed cells. An equivalent amount of ^111^In chloride resulted in a twofold decrease from 100 ± 5% to 46 ± 4% only. Combination indices were 1.08 and 1.49 at 3 MBq/μg at 1 and 2 μM, respectively, but ranged from 0.66 to 0.14 for 6 and 9 MBq/μg at 1 and 2 μM, indicating superadditivity between ^111^In and F3.

### *In vivo *biodistribution of ^111^In-BnDTPA-F3

**Figure 5:**
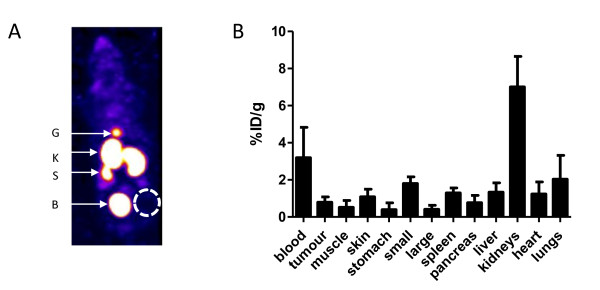
***In vivo *biodistribution of ^111^In-BnDTPA-F3**. (**A**) Representative SPECT image of the distribution of ^111^In-BnDTPA-F3 in 231-H2N xenograft-bearing mice, 3-h post-intravenous injection. G, gall bladder; K, kidneys; S, spleen; B, bladder. (**B**) Biodistribution of ^111^In-BnDTPA-F3, 3-h post-intravenous injection. Results are expressed as the average percentage of the injected dose per gram (%ID/g) of tissue ± SD of the three mice.

A representative SPECT maximum intensity projection, 3 h after intravenous injection of 3 μg of ^111^In-BnDTPA-F3 (6 MBq/μg), is shown in Figure 5A. The biodistribution of ^111^In-BnDTPA-F3 was determined 3 h after i.v. injection of 3 μg (6 MBq/μg). The results are summarized in Figure 5B. ^111^In-BnDTPA-F3 was mainly taken up in the kidneys (7.0 ± 1.6%ID/g). ^111^In-BnDTPA-F3 concentration in the blood was relatively high for a peptide of this size (3.2 ± 1.6%ID/g). Tumor uptake was modest (0.80 ± 0.28%ID/g).

### Tumor growth inhibition

**Figure 6:**
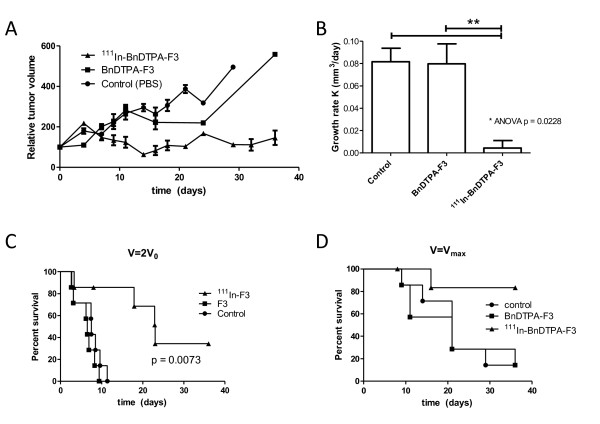
***In vivo *xenograft growth inhibition by ^111^In-BnDTPA-F3**. Mice, bearing 231-H2N xenografts, were injected intravenously with PBS, BnDTPA-F3, or ^111^In-BnDTPA-F3. Tumor size was measured twice weekly by a caliper. (**A**) Normalized tumor volume. (**B**) Growth rate (*K*) in mm^3 ^per day. Results are expressed as average growth rates of tumors in seven mice ± SEM. **p = 0.0031. (**C**) Kaplan-Meier survival curves generated using the time tumors reached twice the size at the beginning of the study (*V *=*2V*_0_). (**D**) Kaplan-Meier survival curves. Mice were killed when the tumor reached its maximally allowed volume (*V *=*V*_max_).

The 231-H2N xenograft tumors in mice that received three weekly doses of ^111^In-BnDTPA-F3 (3 μg, 6 MBq/μg) grew significantly slower compared with those in mice that received cold, unlabeled BnDTPA-F3 or PBS control (growth rate = 0.0043 ± 0.0061, 0.080 ± 0.019, and 0.082 ± 0.013 mm^3^/day, respectively; *p *= 0.0031) (Figure 6A, B). Kaplan-Meier curves showed a significant difference in time for the tumor to grow twice its original volume (*p *= 0.0073) (Figure 6C) as well as survival time (*p *= 0.0174) (Figure 6D).

## Discussion

Auger electron radiation therapy has been investigated extensively for the eradication of cancer, *in vitro *and *in vivo *in pre-clinical models [8]. In this report, an Auger electron-emitting, ^111^In-labeled radiopharmaceutical, based on the HMGN2-protein-derived F3 peptide is presented. It was shown that FITC-F3 binds to 231-H2N cells, internalizes, and translocates to the nucleus (Figure 1), confirming previously reported data [11,16,18]. This not only proves F3 internalization in the model cell line used here, 231-H2N, but furthermore suggests that when radiolabeled the same peptide might also be taken up into these cells and their nuclei. ^111^In-BnDTPA-F3 also internalizes into these cells and is taken up into the nucleus (Figure 2) in a nucleolin-dependent fashion, as indicated in experiments in which cold, unlabeled F3 peptide and anti-nucleolin antibodies were used to block uptake. As suggested by Henke et al., blockage of internalization by the anti-nucleolin antibody, ZN004, is most likely not due to steric hindrance but to antibody-dependent blockage of nucleolin internalization [13]. Even though only 0.15% of the total added ^111^In accumulates in the nuclei of 231-H2N cells, the nuclear uptake of ^111^In-BnDTPA-F3 was sufficient to result in the formation of DNA dsb. This dose-dependent DNA damage formation occurs rapidly as already 2 h after addition of ^111^In-BnDTPA-F3 γH2AX foci could be observed. The induction of DNA dsb in turn led to a dose-dependent decrease in clonogenic survival (Figure 4A). The effect of increasing specific activity shown in Figure 4B further corroborates the finding that reduced clonogenic survival is caused by the radiotoxicity of ^111^In. *In vivo*, after intravenous injection in mice bearing a 231-H2N xenograft, overall tumor uptake of ^111^In-BnDTPA-F3 is modest, but the growth of the tumor can be slowed significantly by ^111^In-BnDTPA-F3, but not unlabeled BnDTPA-F3.

Drecoll et al. reported extensive and rapid nuclear localization of ^213^Bi-DTPA-(F3)_2 _in MDA-MB-435 cells [19], while the results in this paper show that cellular internalization of ^111^In-BnDTPA-F3, albeit in a different cell line, was low and increased steadily up to 2 h. Nuclear internalization of ^111^In-DTPA-F3 was 37% of the total cellular uptake in 231-H2N cells, whereas nucleus-associated ^213^Bi exceeded 75% of cell-bound ^213^Bi. Although these studies were performed in different cells, this discrepancy in nuclear uptake might be explained by the bi-valency of the ^213^Bi compound (monomer vs. dimer).

Given the relatively longer pathlength of alpha particles (50 to 100 μm), there is no requirement for ^213^Bi-DTPA-(F3)_2 _to internalize into the tumor cells; association with the membrane of tumor cells is sufficient. In contrast, the Auger electrons emitted by ^111^In have a very short pathlength; most of the energy is deposited in the first few nanometers [6]. Therefore, it is hypothesized that nuclear localization is necessary for Auger electron therapy to be effective. It was shown here that ^111^In-BnDTPA-F3 does not internalize into the cells in large quantities but, nevertheless, results in a radiation absorbed dose to tumor cell nuclei of 10.8 Gy, which is comparable to other Auger electron-emitting agents [24]. This is further corroborated by the presence of γH2AX foci in cells, shortly after exposure to ^111^In-BnDTPA-F3, and by the linear correlation between specific activity and gH2AX foci induction. Taken together, these results suggest that ^111^In-BnDTPA-F3 causes nucleolin-specific, ^111^In-specific radiocytotoxicity in 231-H2N cells.

The biodistribution of ^111^In-BnDTPA-F3 in a xenograft-bearing mouse, 3 h after intravenous injection, was comparable to that of ^68^Ga-DOTA-F3 as it also showed high kidney uptake and low liver signal, indicating that the excretion of this small labeled peptide occurs for the most part through a renal clearance. However, tumor uptake of ^111^In-BnDTPA-F3 was much lower (1.0%ID/g) compared with that of ^213^Bi-(F3)_2 _(32%ID/g). This may be attributed to the locoregional (intraperitoneal) administration used for ^213^Bi-(F3)_2 _or to the bivalency of the ^213^Bi-compound causing increased avidity. Another possibility is that the relatively large size of ^213^Bi-(F3)_2 _could prolong its global retention. Interestingly, in a recent report by Bhojani et al., the uptake of a ^125^I-labeled F3 peptide in MDA-MB-435 xenografts was much closer to that of ^111^In-BnDTPA-F3 than ^213^Bi-(F3)_2_, being 1.05% ID/g at 30 min following i.v. injection [17]. Most likely, the rapid renal clearance of ^125^I-F3 and ^111^In-BnDTPA-F3 following intravenous injection limits the bioavailability of F3. Although no saturation of ^111^In-BnDTPA-F3 cell association was observed *in vitro *[see Figure 1 in Additional file 1], it is also possible that saturation of the tumor cell membranar nucleolin by F3 peptides is responsible for the modest tumor uptake (3 and 19 μg of ^111^In-BnDTPA-F3 and ^125^I-F3 were injected, respectively, compared to 0.33 μg of ^213^Bi-(F3)_2_).

Although tumor uptake of ^111^In-BnDTPA-F3 was modest, i.v. administration of ^111^In-BnDTPA-F3 significantly inhibited the growth of 231-H2N xenograft tumors. Given the extremely high dose deposition around a decaying ^111^In nuclide (up to 5,000 Gy absorbed in the 10-nm range), there is no necessity for a high tumor uptake, as long as the intracellular trafficking of ^111^In ensures its close proximity to densely packed DNA, as in the nucleolus. Interestingly, it has recently been highlighted in the literature that nucleolar function is particularly sensitive to cellular stress including that caused by UV and ionizing radiation [27,28]. In particular, it has emerged that the nucleolus is directly involved in p53 regulation so that nucleolar disruption may be associated with changes in cell cycle and apoptosis [29]. It is, therefore, intriguing to speculate that even modest accumulation of radioactivity in the nucleoli, by causing disruption of stress-response co-ordination, could have a disproportionately detrimental effect on cell survival. It has been reported that intravenously injected FITC-F3 localized in CD31 positive tumor vessels [9,10,16,18]. Therefore, it is possible that some of the anti-tumor effect of ^111^In-BnDTPA-F3 results from targeting of the tumor vasculature. Taken together, these results suggest that ^111^In-BnDTPA-F3 is capable of causing significant tumor growth inhibition.

## Conclusion

^111^In-BnDTPA-F3 is an Auger electron-emitting radiopharmaceutical that internalizes in tumor cells where it accumulates in the nucleus and, in particular, in the nucleoli, causing decreased clonogenic survival *in vitro *and tumor growth inhibition *in vivo*.

## Competing interests

The authors declare that they have no competing interests.

## Authors' contributions

BC and KV conceived the study and participated in its design and coordination. AW carried out the DNA damage studies. CW performed immunocytochemistry. BC, VK, and SS designed and carried out *in vivo *experiments. All authors read and approved the final manuscript.

## Supplementary Material

Additional file 1 **Internalization and nuclear localization of ^111^In-BnDTPA-F3**. The 231-H2N cells were seeded per well in 24-well plates, and ^111^In-BnDTPA-F3 was then added (6 to 1,360 μg/mL in 200 μL DMEM, 6 MBq/mg) for 2 h at 37°C. Cells were washed three times with PBS, and cell-associated radioactivity was counted. Click here for file
