# Supplementary figures and images for: 111In-BnDTPA-F3: an Auger electron-emitting radiotherapeutic agent that targets nucleolin
Source: EJNMMI Res. 2012 Feb 20;2:9. doi: 10.1186/2191-219X-2-9 (PMC3298710; doi:10.1186/2191-219X-2-9)

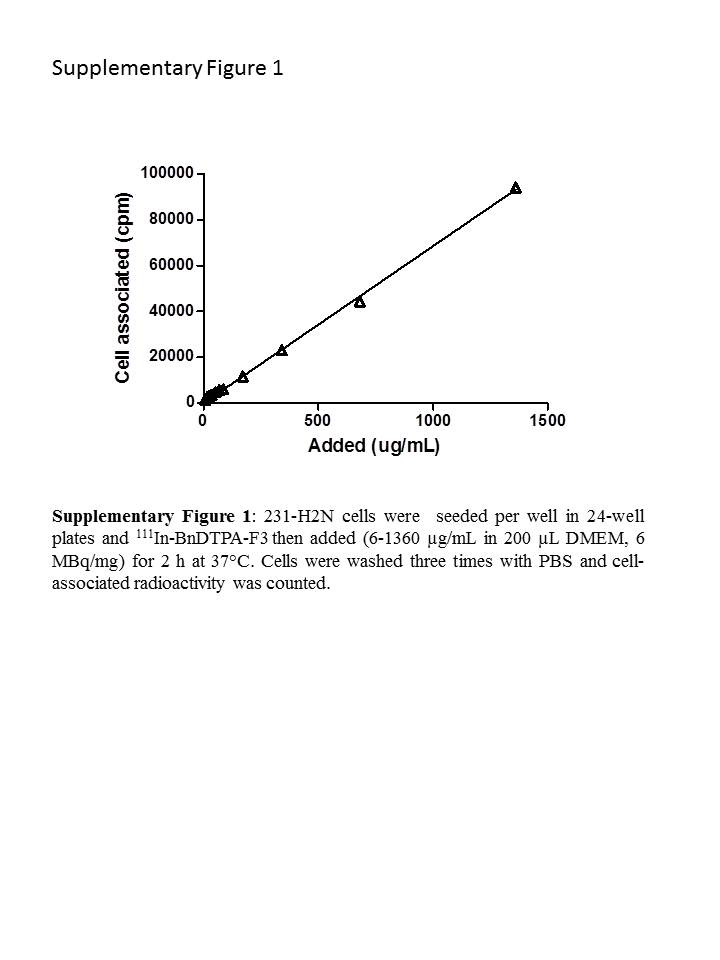

Supplement: Additional file 1 — Internalization and nuclear localization of 111In-BnDTPA-F3. The 231-H2N cells were seeded per well in 24-well plates, and 111In-BnDTPA-F3 was then added (6 to 1,360 μg/mL in 200 μL DMEM, 6 MBq/mg) for 2 h at 37°C. Cells were washed three times with PBS, and cell-associated radioactivity was counted. [file 2191-219X-2-9-S1.JPEG]
